# Quantifying Quality of Life and Disability of Patients with Advanced Schistosomiasis Japonica

**DOI:** 10.1371/journal.pntd.0000966

**Published:** 2011-02-15

**Authors:** Tie-Wu Jia, Jürg Utzinger, Yao Deng, Kun Yang, Yi-Yi Li, Jin-Huan Zhu, Charles H. King, Xiao-Nong Zhou

**Affiliations:** 1 Key Laboratory on Biology of Parasites and Vectors, MOH, WHO Collaborating Center on Malaria, Schistosomiasis and Filariasis, National Institute of Parasitic Diseases, Chinese Center for Disease Control and Prevention, Shanghai, People's Republic of China; 2 Department of Public Health and Epidemiology, Swiss Tropical and Public Health Institute, Basel, Switzerland; 3 University of Basel, Basel, Switzerland; 4 Jiangsu Institute of Parasitic Diseases, Wuxi, People's Republic of China; 5 Hunan Institute of Parasitic Diseases, Yueyang, People's Republic of China; 6 Center for Global Health and Diseases, Case Western Reserve University School of Medicine, Cleveland, Ohio, United States of America; University of Kelaniya, Sri Lanka

## Abstract

**Background:**

The Chinese government lists advanced schistosomiasis as a leading healthcare priority due to its serious health and economic impacts, yet it has not been included in the estimates of schistosomiasis burden in the Global Burden of Disease (GBD) study. Therefore, the quality of life and disability weight (DW) for the advanced cases of schistosomiasis japonica have to be taken into account in the re-estimation of burden of disease due to schistosomiasis.

**Methodology/Principal Findings:**

A patient-based quality-of-life evaluation was performed for advanced schistosomiasis japonica. Suspected or officially registered advanced cases in a *Schistosoma japonicum*-hyperendemic county of the People's Republic of China (P.R. China) were screened using a short questionnaire and physical examination. Disability and morbidity were assessed in confirmed cases, using the European quality of life questionnaire with an additional cognitive dimension (known as the “EQ-5D plus”), ultrasonography, and laboratory testing. The age-specific DW of advanced schistosomiasis japonica was estimated based on patients' self-rated health scores on the visual analogue scale of the questionnaire. The relationships between health status, morbidity and DW were explored using multivariate regression models. Of 506 candidates, 215 cases were confirmed as advanced schistosomiasis japonica and evaluated. Most of the patients reported impairments in at least one health dimension, such as pain or discomfort (90.7%), usual activities (87.9%), and anxiety or depression (80.9%). The overall DW was 0.447, and age-specific DWs ranged from 0.378 among individuals aged 30–44 years to 0.510 among the elderly aged ≥60 years. DWs are positively associated with loss of work capacity, psychological abnormality, ascites, and active hepatitis B virus, while splenectomy and high albumin were protective factors for quality of life.

**Conclusions/Significance:**

These patient-preference disability estimates could provide updated data for a revision of the GBD, as well as for evidence-based decision-making in P.R. China's national schistosomiasis control program.

## Author Summary

Advanced schistosomiasis japonica, an extreme form of chronic schistosomiasis that occurs in Asia, is more serious than the advanced hepatosplenic disease of schistosomiasis encountered in Africa and the Americas. The advanced schistosomiasis japonica is a chronic disabling condition associated with portal hypertension, splenomegaly, ascites, and gastro-oesophageal variceal bleeding, or with severe growth retardation or granulomatous disease of the large intestine. However, the actual disability caused by advanced schistosomiasis japonica is unknown. We carried out a patient-based quality-of-life evaluation employing a standardized and widely used questionnaire (known as “EQ-5D plus”), coupled with ultrasonography and laboratory tests on advanced schistosomiasis japonica cases in a hyperendemic area of China. Among 215 confirmed cases of advanced schistosomiasis japonica, we found an overall disability weight of 0.447 with age-specific weights ranging from 0.378 to 0.510. Importantly, advanced schistosomiasis japonica is not only associated with heavy disability weights, but also with high morbidity and poor self-reported quality of life. Our results provide valuable data for the current revision of the Global Burden of Disease (GBD) study, as well as for evidence-based decision-making in China's national schistosomiasis control program.

## Introduction

Schistosomiasis, caused by infection with trematode blood flukes of the genus *Schistosoma*, is one of the world's most important helminth infections in terms of the global burden of human morbidity and mortality [1], [2]. Advanced, or late-stage schistosomiasis japonica can be regarded as an extreme form of chronic Asian schistosomiasis, one that is more serious than the advanced hepatosplenic disease of *Schistosoma mansoni* infection found in Africa and the Americas. In the People's Republic of China (P.R. China), advanced schistosomiasis is a chronic disabling condition associated with portal hypertension, splenomegaly, ascites, and gastro-oesophageal variceal bleeding, or with severe growth retardation or granulomatous disease of the large intestine. In this country, advanced cases are registered and managed independently from patients with general chronic schistosomiasis.

Advanced schistosomiasis japonica is much more common in highly endemic areas, because repeated, heavy exposure to cercariae means that early-stage chronic cases may not be effectively treated in routine control programs. The eggs of *S. japonicum* retained in the intestine and liver tissue stimulate a granulomatous response, leading to continuous fibrosis of the periportal tissue and developing a pipestem fibrosis. Although down-modulation of the granulomatous response, which could prevent excessive chronic morbidity [3] after 2–5 years or more, parasite-induced periportal fibrosis may progress to cause obstruction of the portal vessels and damage to the liver parenchyma, leading to development of advanced schistosomiasis [4]. Mortality eventually results from bleeding of the upper gastrointestinal tract, spontaneous bacterial peritonitis, and hepatic failure, among others [5], [6]. Based on its major symptoms, advanced schistosomiasis japonica in P.R. China represents a common, serious health burden, and has been classified into four clinical sub-types, namely (i) ascites, (ii) megalosplenia, (iii) colonic tumoroid proliferation, and (iv) dwarfism [5], [6].

In the 1950s, it was estimated that 5–10% of the *S. japonicum*-infected individuals in areas highly endemic for schistosomiasis would develop to the advanced stages of disease. At that time, there were approximately 500,000 advanced cases in P.R. China [4]. Terms like “villages of widows” and “villages where all is dead” were used to describe the devastating impact of schistosomiasis across southern P.R. China [7]. Over the past 60 years, implementation of integrated control approaches has succeeded in greatly reducing the burden due to schistosomiasis in P.R. China and, at present, dwarfism and colonic tumoroid proliferation are rarely found [8], [9]. However, ascites and megalosplenia are still common, typically in foci of high transmission intensity, but also in areas where the transmission of schistosomiasis has been controlled and interrupted for several decades, such as Shanghai municipality and Zhejiang province [10], [11]. By the end of 2008, a total of 412,927 cases of schistosomiasis were found in P.R. China, and among them, 30,030 (7.3%) suffered from the advanced form of chronic schistosomiasis japonica [12].

Schistosomiasis represents a serious, but under-recognized, disease burden in many developing countries [13]. Unfortunately, in the World Health Organization/World Bank Global Burden of Disease (GBD) study, active schistosome infection was the only health state evaluated in the assessment of schistosomiasis-associated disease burden. Based on older, unfounded notions of ‘minimal to absent symptomatology’ in uncomplicated chronic schistosomiais, the GBD assigned a schistosomiasis disability weight (DW) of 0.005 (on a scale from 0 (no disability) to 1 (death)) for school-aged children, and 0.006 for those aged ≥15 years [14]. More recent studies suggest, however that this is a serious underestimation of the ‘true’ disability due to schistosomiasis [15]–[18].

In the mid-2004s, we successfully introduced a patient-based evaluation, the so-called EQ-5D plus questionnaire, as a measure of health-related quality of life to assess the disability impact of early stage chronic schistosomiasis japonica [16]. Of note, the EQ-5D plus questionnaire had been widely used in different settings for measuring population health status [19]–[21]. We concluded that the overall DW for early stage chronic schistosomiasis was, on average, 0.191 and age-specific weights ranged from 0.095 (children aged 5–14 years) to 0.246 (elderly aged ≥60 years) [16]. In 2006, another independent study carried out in Hubei province on the basis of disability weighting definitions of the GBD study [22] obtained a similar DW of 0.122 [23]. These estimates supported findings from two meta-analyses performed by King and colleagues [15], [18], and a disability-adjusted life year (DALY)-based life-path model developed by Finkelstein *et al.* [17]. However, these studies were all limited to a general valuation of the disabling sequelae of chronic schistosome infection. In view of the considerable magnitude and fatal clinical outcomes of advanced schistosomiasis japonica, and relatively independent case management in P.R. China, we considered it important to explore the independent contribution of advanced schistosomiasis japonica to the national and global disease burden. Although there are many studies on the topic of advanced schistosomiasis, very few studies have attempted to assess the patients' disability in terms of overall quality of life [10], [24].

## Methods

### Study area and population

This study was carried out between October 2007 and January 2008 in Hanshou county, Hunan province, which is hyperendemic for *S. japonicum*, and where a considerable number of patients with advanced schistosomiasis still reside. All suspected or officially registered advanced schistosomiasis cases in Hanshou county were eligible for enrolment. A short questionnaire was administrated and a physical examination was carried out to screen for advanced cases. Those who had reached ‘clinical cure’, or those who were clearly co-morbid with other serious diseases such as tuberculosis, diabetes, cardiopathy, nephropathy, and hepatic cirrhosis, were excluded from the present study.

According to the national standardized diagnostic criteria for schistosomiasis (WS261-2006), the inclusion criteria for advanced schistosomiasis cases were as follows: (i) repeated or long-term exposure to cercaria-infested water or a history of chemotherapy against schistosomiasis; (ii) positive serological test (enzyme-linked immunosorbent assay (ELISA)); and (iii) portal hypertension syndrome resulting from hepatic fibrosis, e.g., ascites, splenomegaly reaching Hackett grade 3 or higher, or splenomegaly of Hackett grade 2 but with hypersplenism, dilatation of oesophageal or gastric varices, or upper gastrointestinal bleeding. Based on the major symptoms, advanced schistosomiasis is classified into four clinical types, namely (i) ascites; (ii) megalosplenia; (iii) colonic tumoroid proliferation; and (iv) dwarfism [5], [6], [25], each type of which was qualified for the study. Those subjects who had undergone splenectomy but who had persistent signs and symptoms of abdominal pain, diarrhea, or weakness (i.e., not having reached a status of ‘clinical cure’) were also eligible for inclusion.

### Questionnaire and diagnostic procedures

The study subjects were first interviewed using a standardized and pre-tested questionnaire and, subsequently, participants were subjected to a physical examination, ultrasonography, and laboratory testing. Regarding interviews, an individual questionnaire was used to obtain information on sociodemographic variables (i.e., age, sex, educational attainment, and occupation), exposure to cercaria-infested water, history of anti-schistosomal treatment (including chemotherapy or splenectomy), and self-reported symptoms and signs during the past 12 months, including fatigue, anorexia, abdominal distension or pain, diarrhea, blood in the stool, as well as any partial or complete loss of working capacity. Additionally, the EQ-5D plus questionnaire was employed to assess the respondents' health-related quality of life in six relevant dimensions, namely (i) mobility; (ii) self-care; (iii) participation in usual activities; (iv) the presence of pain or discomfort; (v) the presence of anxiety or depression; and (vi) altered cognition. For each dimension, three possible outcomes were considered: no problems, moderate problems, or extreme problems. The questionnaire also included a 20 cm visual analogue scale (VAS) for the self-rated valuation of patient's own general health status on a continuous scale from best imaginable (100) to the worst imaginable (0) [20], [21], [26], [27]. This VAS was subsequently used to derive the individual subject's disability score (see details below).

Abdominal ultrasonography was performed with the subject in a fasting state (i.e., no food intake 4 hours prior to examination). Subjects were horizontally-positioned and organometric measurements were taken during relaxed inhalation. Pathology was graded according to standardized criteria [25]. Hepatic fibrosis was graded from 0 (normal) to grade III (severe). Hepatomegaly was defined as a protrusion of the liver of >3 cm under the xiphoid process or palpable (>0 cm) under the right costal margin at the midclavicular line. Inner diameter of main portal vein was measured by ultrasonography and compared to established normal values (10.9±1.1 mm for individuals aged 30–39 years, 11.1±1.1 mm for people aged 40–49 years, 10.7±1.2 mm for individuals aged 50–59 years, and 10.6±0.9 mm for elderly aged ≥60 years) [25]. Splenomegaly was determined by physical examination using the Hackett classification (grade 1–5). The presence of ascites was confirmed by ultrasonography and graded clinically as none, mild, moderate, or severe [6].

With regard to laboratory testing, a blood sample was obtained for measurement of hepatitis B virus-related antigens and antibodies, anti-schistosomal antibody, blood hemoglobin, and serum albumin concentration.

**Figure 1:**
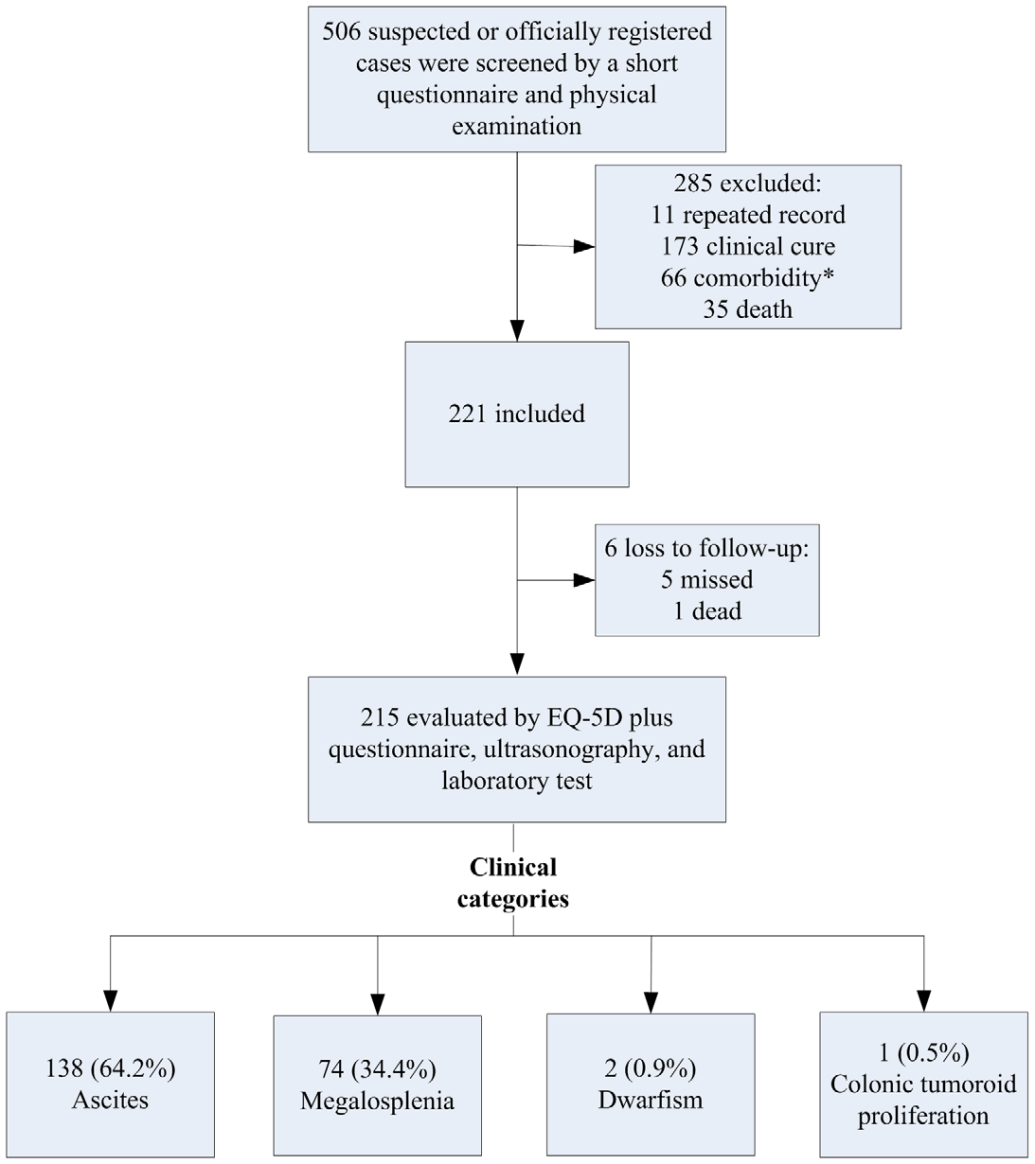
Flow chart, detailing how many advanced cases of schistosomiasis japonica were included and excluded in the study. Of note, there were 66 advanced cases of schistosomiasis japonica with comorbidities, such as diabetes, cardiopathy, nephropathy, and hepatic cirrhosis.

Although participants were not specifically examined for other diseases, those with clinical symptoms and signs clearly attributable to conditions other than schistosomiasis were excluded from the study. The number of advanced cases of schistosomiasis japonica were included and excluded in the study with the study flow chart showed in Figure 1.

### Statistical analysis

An anonymous database was created and all entries double-checked. Statistical analyses were performed using SAS software version 8.1 (SAS Institute; Cary, USA). The χ^2^ test was used to examine differences between categories, and the Cochran-Mantel-Haenszel χ^2^ test was employed to explore linear associations between outcome variables and age. The DW for each individual was computed, based on the self-rated health score on the linear VAS using the following formula: *DW* = 1−(*VAS* score / 100), where *DW* is the estimated disability weight for that subject.

The mean DWs were also calculated for subgroups stratified by age or clinical type. Analysis of variance (ANOVA) was used to test for differences in mean DWs among all groups, and the Bonferroni *t*-test was used for pairwise comparisons. Two multivariate regression models were developed to explore the morbidity indicators associated with DW. Model 1 assessed the relationship between age, exposure, the separate dimensions of the EQ-5D plus questionnaire, and the subject's DW outcome, whilst model 2 assessed the association between morbidity indicators, socioeconomic status, and DW. Independent variables showing no statistical significance (*P*>0.05) were removed by a backward stepwise elimination procedure. Age, occupation, educational attainment, hepatic fibrosis, loss of work capacity, and ascites were specified as categorical variables, with a designated reference category and a set of contrasted dummy variables.

### Ethics statement

The study protocol was approved by the institutional review board of the National Institute of Parasitic Diseases, Chinese Center for Disease Control and Prevention in Shanghai, and the WHO Research Ethics Review Committee. The objectives, procedures, and potential risks were explained to all participants. Written informed consent was also obtained from each participant or a literate relative. Individuals who were seropositive for anti-schistosomal antibody were treated with praziquantel, free of charge, according to Chinese national guidelines for schistosomiasis control.

## Results

### Characteristics of study cohort

During the initial screening, a total of 506 suspected or officially registered cases were examined. Among them, 221 were confirmed as advanced schistosomiasis japonica cases and 215 were evaluated. Males represented 71.6% (154/215) of the total study cohort, and 80.5% (173/215) were older than 44 years (mean age: 57.1 years, standard deviation: 12.6 years; range: 30–80 years).

### Clinical findings

The observed proportions of the different clinical categories of advanced schistosomiasis were: 64.2% (138/215) for ascites (type I), 34.4% (74/215) for megalosplenia (type II), 0.5% (1/215) for colonic tumoroid proliferation (type III), and 0.9% (2/215) for dwarfism (type IV). There was no significant difference in the distribution of clinical categories between males and females (χ^2^ = 0.295, *P* = 0.587).

Table 1 shows the number and percentage of patients having schistosomiasis-related clinical symptoms or signs, stratified by age, among all the enrolled patients with advanced schistosomiasis. There were no statistically significant differences in the rates of history of prior treatment, fatigue, anorexia, abdominal pain, diarrhea, or blood in the stool among the different age groups. There was no statistically significant difference in the rate of splenectomy between those aged 30–44 years and those aged 45–59 years (*P*>0.05), but there was a statistically significant difference between those aged 30–59 and those aged ≥60 years (*P*<0.001). Overall, 84.9% (62/73) of the patients who had undergone splenectomy were below the age of 60 years. The prevalence of ascites increased with age from 23.8% (10/42) among those aged 30–44 years to 36.3% (47/101) among those aged ≥60 years (*P*<0.01).

**Table 1:** Clinical symptoms or signs in advanced cases of schistosomiasis japonica, stratified by age group (n = 215).

Age group (years)	No. of cases	Number (%) of clinical symptoms and signs									
		Splenectomy^a^	History of treatment	Fatigue	Anorexia	Abdominal distension^b^	Abdominal pain	Diarrhea	Blood in the stool	Impaired work capacity^c^	Ascites^d^
30–44	42	22 (52.4)	36 (85.7)	39 (92.9)	30 (71.4)	30 (71.4)	18 (42.9)	13 (31.0)	6 (14.3)	32 (76.2)	10 (23.8)
45–59	72	40 (55.6)	67 (93.1)	69 (95.8)	58 (80.6)	48 (66.7)	39 (54.2)	26 (36.1)	7 (9.7)	66 (91.7)	21 (29.2)
≥60	101	11 (10.9)	88 (87.1)	96 (95.1)	82 (81.2)	91 (90.1)	63 (62.4)	45 (44.6)	22 (21.8)	100 (99.0)	47 (46.5)
All	215	73 (34.0)	191 (88.8)	204 (94.9)	170 (79.1)	169 (78.6)	120 (55.8)	84 (39.1)	35 (16.3)	198 (92.1)	78 (36.3)

a Significant difference between age groups for general association (*P*<0.001), without statistically significant differences in the rate of splenectomy between individuals aged 30–44 years and those aged 45–59 years (*P*>0.05), but with statistically significant difference between the age groups 30–59 years and ≥60 years (*P*<0.001).

b Significant difference between age groups for general association (*P*<0.001) and a linear association between abdominal distension and age (*P*<0.01).

c Significant difference between age groups for general association (*P*<0.001) and a linear association between work capacity and age (*P*<0.001).

d Significant difference between age groups for general association (*P*<0.05) and a linear association between ascites and age (*P*<0.01).

The prevalence of self-reported impairment of work capacity (partial or complete loss) also showed a strong increase with age, rising from 76.2% (32/42) among those aged 30–44 years to 99.0% (100/101) among those aged ≥60 years (*P*<0.001). Among all enrolled subjects, 29.8% (64/215) had complete loss of work capacity (by age group: 30–44 years, seven cases; 45–59 years, nine cases; and ≥60 years, 48 cases), whilst an additional 62.3% (134/215) of subjects had partial loss of work capacity (by age group: 30–44 years, 25 cases; 45–59 years, 57 cases; and ≥60 years, 52 cases).

### Laboratory and ultrasonographic findings

Table 2 shows the results of laboratory and ultrasonography testing for the study subjects. The mean hemoglobin level was 9.73 g/dl (standard deviation (SD) = 2.11 g/dl) among males and 8.83 g/dl (SD = 1.68 g/dl) among females. The hemoglobin level of males was 0.89 g/dl higher than that of females (*F* value = 7.61, *P*<0.01). In comparison to normal levels (12–16 g/dl for males, and 11–15 g/dl for females), the mean hemoglobin of males was 2.27 g/dl lower than average (*t* = −13.04, *P*<0.001), while the mean hemoglobin of females was found to be 2.17 g/dl lower than average (*t* = −9.31, *P*<0.001). The measured albumin levels for the study subjects were within normal limits (35–55 g/l; *t* = 1.81, *P* = 0.072) and there were no significant difference among age groups in albumin levels (*F* = 2.98, *P* = 0.053). The rates of circulating HBsAg and anti-HBc antibody positivity were relatively high in the study cohort, but circulating HBeAg was observed in only 2.0% (4/198). An inverse association with age was found for hepatomegaly; the prevalence declined from 46.0% (17/37) among those aged 30–44 years to 25.8% (25/97) among those aged ≥60 years (*P*<0.05).

**Table 2:** Physical and ultrasonographic abnormalities in advanced cases of schistosomiasis japonica, stratified by age group (n = 198).

Age (years)	No.of cases	Mean haemoglobin (SD) in g/dl^a^	Mean albumin (SD) in g/l	Mean inner diameter of portal vein (SD) in mm^b^	HBsAg^c^ (%)	Anti-HBc^d^ (%)	HBeAg^e^ (%)	Hepatomegaly^f^ (%)	Hepatic fibrosis (%)			
									Grade I	Grade II	Grade III	Total
30–44	37	10.27 (2.08)	37.5 (6.7)	15.3 (5.6) (n = 36)	14 (37.8)	13 (35.1)	1 (2.7)	17 (46.0)	11 (29.7)	2 (5.4)	23 (62.1)	36 (97.3)
45–59	64	9.88 (1.87)	36.4 (5.4)	14.8 (1.9) (n = 61)	30 (46.9)	27 (42.2)	1 (1.6)	22 (34.4)	25 (39.1)	3 (4.7)	35 (54.7)	63 (98.4)
≥ 60	97	8.94 (1.99)	34.8 (6.3)	15.1 (1.6) (n = 95)	21 (21.7)	19 (19.6)	2 (2.1)	25 (25.8)	22 (22.7)	7 (7.2)	63 (65.0)	92 (94.9)
All	198	9.49 (2.04)	35.8 (6.2)	15.0 (2.9) (n = 192)	65 (32.8)	59 (29.8)	4 (2.0)	64 (32.3)	58 (29.3)	12 (6.1)	121 (61.1)	191 (96.5)

From the 215 cases with advanced schistosomiasis japonica, 17 observations were excluded owing to missing values.

a Analysis of variance was performed for means of hemoglobin (*F* value = 8.03, *P*<0.001). Bonferroni *t*-test was performed for comparisons between age groups. The mean of hemoglobin of those aged ≥60 years was significantly different from those aged 30–44 years and those aged 45–59 years at a level of 5%, and there were no statistical significance between those aged 30–44 years old and those aged 45–59 years at a level of 5%.

b The mean of inner diameter of portal vein was 3.9 mm (SD = 2.9 mm) larger than the normal value (10.6–11.1 mm for those aged ≥30 years; *t* = 18.92, *P*<0.001) and there was no significant difference of means between age groups (*F* = 0.32, *P* = 0.726).

c Hepatitis B surface antigen. Significant difference between age groups for general association (*P*<0.01).

d Specific antibody to hepatitis B core antigen. Significant difference between age groups for general association (*P*<0.01).

e Hepatitis B antigen appearing during weeks 3 to 6 indicates an acute active infection at the peak infectious period, and means that the patient is infectious. Persistence of this virological marker beyond 10 weeks shows progression to chronic infection and infectiousness.

f A linear association between hepatomegaly and age (*P*<0.05).

The mean inner diameter of the main portal vein was 3.9 mm (SD = 2.9 mm) larger than normal values (10.6–11.1 mm for those aged ≥30 years; *t* = 18.92, *P*<0.001) with no significant difference among age groups (*F* = 0.32, *P* = 0.726). Hepatic fibrosis was detected by 96.5% (191/198) of the study subjects, with 6.1% (12/198) having grade II, and 61.1% (121/198) having grade III fibrosis. We did not observe a significant difference in the distribution of hepatic fibrosis severity scores among the different age groups (*P*>0.05).

### Self-rated quality of life

The results obtained through the EQ-5D plus questionnaire are summarized in Tables 3 and 4. Almost all the patients with advanced schistosomiasis japonica complained of some impairment. Moderate impairment was reported by 54.4% (117/205), extreme impairment by 41.9% (90/215), with the highest prevalence of reported disability found in the pain or discomfort dimension (90.7%, 195/215). Impairment in performance of usual activities is a typical sequela of advanced schistosomiasis and this form of disability was common in our study cohort (87.9%, 189/215). Impaired mobility and self-care are considered more extreme forms of disability, and these were reported fairly frequently by the advanced schistosomiasis patients (31.6% (68/215) and 30.7% (66/215) of subjects, respectively). The prevalence of impairment in each of the six dimensions increased with age (*P*<0.001), such that, among subjects ≥60 years, 100% (101/101) reported impairment in at least one dimension of performance.

**Table 3:** Results obtained from EQ-5D plus questionnaire in 215 patients with advanced schistosomiasis japonica, stratified by degree of health problem.

Dimension	Degree of health problem, number (%)			Any problem
	None	Moderate	Extreme	
Mobility	147 (68.4)	64 (29.8)	4 (1.9)	68 (31.6)
Self-care	149 (69.3)	58 (27.0)	8 (3.7)	66 (30.7)
Usual activities	26 (12.1)	127 (59.1)	62 (28.8)	189 (87.9)
Pain or discomfort	20 (9.3)	179 (83.3)	16 (7.4)	195 (90.7)
Anxiety or depression	41 (19.1)	118 (54.9)	56 (26.1)	174 (80.9)
Cognition	72 (33.5)	89 (41.4)	54 (25.1)	143 (66.5)
Any dimension	8 (3.7)	117 (54.4)	90 (41.9)	207 (96.3)

**Table 4:** Results obtained from EQ-5D plus questionnaire in patients with advanced schistosomiasis japonica, stratified by age group.

Age (years)	No. of cases	Dimension, number (%)						
		Mobility^a^	Self-care^b^	Usual activities^c^	Pain or discomfort^b^	Anxiety or depression^c^	Cognition^a^	Any dimension^c^
30–44	42	7 (16.7)	10 (23.8)	32 (76.2)	33 (78.6)	28 (66.7)	16 (38.1)	36 (85.7)
45–59	72	13 (33.5)	14 (19.4)	59 (81.9)	65 (90.3)	53 (73.6)	40 (55.6)	70 (97.2)
≥60	101	48 (47.5)	42 (41.6)	98 (97.0)	97 (96.0)	93 (92.1)	87 (86.1)	101 (100.0)
Total	215	68 (31.6)	66 (30.7)	189 (87.9)	195 (90.7)	174 (80.9)	143 (66.5)	207 (96.3)

a Significant difference between age groups for general association (*P*<0.001) and a linear association between health outcome and age (*P*<0.001).

b Significant difference between age groups for general association (*P*<0.01) and a linear association between health outcome and age (*P*<0.01).

c Significant difference between age groups for general association (*P*<0.001) and a linear association between health outcome and age (*P*<0.001).

### Disability weights

The overall DW derived for all of the subjects with advanced schistosomiasis japonica was 0.447. Age-specific DWs were 0.378, 0.399 and 0.510 for those aged 30–44 years, 45–59 years and ≥60 years, respectively. The difference among the age-specific DWs was found to be highly significant (ANOVA *F* = 17.77, *P*<0.001). Pair-wise comparisons between age groups showed that there were no statistical significance between those aged 30–44 years and those aged 45–59 years, but the mean DW of those aged ≥60 years was significantly higher than those aged 30–44 years and those aged 45–59 years (Table 5).

**Table 5:** Mean disability weights (DWs) of advanced schistosomiasis japonica, stratified by age group.

Age (years)	No. of cases (n = 215)	Mean DW score^a^	SD	95% CI	Minimum score (no. of cases)	Maximum score (no. of cases)
30–44	42	0.378	0.150	0.331–0.425	0.05 (1)	0.70 (2)
45–59	72	0.399	0.138	0.367–0.432	0.00 (1)	0.70 (2)
≥60	101	0.510	0.151	0.480–0.540	0.20 (3)	1.00 (1)
All	215	0.447	0.158	0.426–0.468	0.00 (1)	1.00 (1)

CI, confidence interval; SD, standard deviation.

a Analysis of variance was performed for mean scores (*P*<0.001). Bonferroni *t*-test was performed for comparisons between age groups (α = 0.05). The mean DW of those aged ≥60 years was significantly different from those aged 30–44 years and those aged 45–59 years, and there were no statistical significance between those aged 30–44 years and those aged 45–59 years.

The mean DWs of each clinical type of advanced schistosomiasis japonica are summarized in Table 6. The mean DW was 0.495 for those with ascites (type I) and 0.360 for those with megalosplenia (type II). The DW with ascites was 0.135 (95% confidence interval (CI): 0.093–0.176) higher than for megalosplenia (ANOVA *F* = 41.35, *P*<0.001). There was only one case of colonic tumoroid proliferation and two cases of dwarfism, their associated DWs were calculated at 0.400 (Table 6).

**Table 6:** Mean disability weights (DWs) of advanced schistosomiasis japonica, stratified by clinical type.

Clinical types^a^	No. of cases (n = 215)	Mean DW score	SD	95% CI	Minimum score (no. of cases)	Maximum score (no. of cases)
I	138	0.495^b^	0.152	0.469–0.520	0.05 (1)	1.00 (1)
II	74	0.360^b^	0.133	0.329–0.391	0.00 (1)	0.70 (1)
III	1	0.400	-	-	0.40 (1)	0.40(1)
IV	2	0.400	-	-	0.40 (2)	0.40 (2)
All	215	0.447	0.158	0.426–0.468	0.00 (1)	1.00 (1)

CI, confidence interval; SD, standard deviation.

a Based on the major symptoms, advanced schistosomiasis japonica was classified into four clinical types, namely ascites (I), megalosplenia (II), colonic tumoroid proliferation (III), and dwarfism (IV).

b Analysis of variance was performed for mean scores. The DW of type I was 0.135 (95% CI: 0.093–0.176) higher than type II (ANOVA *F* = 41.35, *P*<0.001).

In assessing the combined impact of different patient attributes on the DW score, we developed two multivariable regression models presented in Tables 7 and 8. In model 1, following stepwise comparison of nested models, we found that the dimensions of self-care and cognition could be removed from the model (*P*>0.05), while the remaining four dimensions of the EQ-5D plus questionnaire each remained positively associated with DW outcomes after adjustment for age, duration of water contact, and the other performance variables (adjusted *R* ^2^ = 0.59, *P*<0.001). The older subjects tended to have a higher DW and those with a longer duration of contact with cercaria-infested water (expressed by the ratio of years of contact with infested water to age) tended to have a lower DW. In model 2, examining other demographic and clinical attributes, the independent variables, including sociodemographic data, the number of previous anti-schistosomal treatments, most reported symptoms, grade of hepatic fibrosis, hepatomegaly, splenomegaly, hemoglobin, and positive HBsAg, failed to predict the DW, and hence were removed from the final model. Splenectomy and a higher albumin level were negatively associated with DWs (adjusted *R* ^2^ = 0.50, *P*<0.05); DW was positively associated with the findings of abdominal distension, abdominal pain, loss of work capacity, ascites, and positive in HBeAg. After multiple adjustments, a complete loss of work capacity and the presence of severe ascites were the strongest predictors of an elevated disability level (highest DW values) (Tables 7 and 8).

**Table 7:** The relationship between the disability weight and the EQ-5D plus questionnaire in multivariate regression model 1.

Parameter	Coefficient	Standard error	t value^a^	*P* value
Intercept	−0.0876	0.0423	−2.07	0.039
Age	0.0017	0.0006	2.79	0.006
Duration of contact with infested water^a^	−0.0897	0.0380	−2.36	0.019
Health status				
Mobility	0.0842	0.0168	5.00	<0.001
Usual activities	0.0333	0.0147	2.27	0.024
Pain or discomfort	0.0607	0.0201	3.02	0.003
Anxiety or depression	0.0827	0.0138	5.99	<0.001

Data derived from 215 patients with advanced schistosomiasis japonica.

a This parameter was expressed by the ratio of years of contact with cercariae-infested water to age.

**Table 8:** The relationship between the disability weight (DW) and related morbidity in multivariate regression model 2.

Parameter	Coefficient	Standard error	t value^a^	*P* value
Intercept	0.3574	0.0579	6.17	<0.001
Splenectomy	−0.0394	0.0184	−2.14	0.034
Abdominal distension	0.0455	0.0218	2.08	0.039
Abdominal pain	0.0571	0.0165	3.46	<0.001
Partial loss of work capacity	0.1075	0.0297	3.63	<0.001
Complete loss of work capacity	0.2186	0.0323	6.78	<0.001
Moderate ascites	0.0847	0.0316	2.68	0.008
Severe ascites	0.2532	0.0774	3.27	0.001
Albumin level	−0.0030	0.0013	−2.32	0.022
Positive of HBeAg	0.1235	0.0545	2.27	0.025

Data are based on 198 patients with advanced schistosomiasis japonica (17 observations had missing values, and hence were omitted from this model).

## Discussion

Although the general impact of schistosome infection has been well reviewed in recent years, there is no special attention paid to advanced hepatosplenic disease due to schistosomiasis in the GBD study [15], [18], [28], [29]. As a late-stage of chronic schistosomiasis, the harms of advanced schistosomiasis are more extensive, intensive and fatal with a protracted course [10], [24], [30], [31]. Undoubtedly, the language gap is an important issue explaining the limited application of the Chinese literature in the updating of the schistosomiasis burden in the GBD study [32], [33]. Moreover, only few studies attempted to assess the patient's disability in terms of overall quality of life [10], [24], [30], [31]. In our preceding investigations, we concluded that the DW of chronic schistosomiasis japonica (early stage) is considerably higher than that put forth by expert opinion, which fed into the original GBD study in the 1990s [16]. The current study clearly shows that advanced schistosomiasis japonica is associated with poor self-reported quality of life, high morbidity, and heavy disability.

As a whole, the overall DW of advanced schistosomiasis japonica is 0.447, 2.34 times higher than that of chronic cases and 4.30 times higher than that of the advanced hepatic disease due to schistosomiasis (DW = 0.104) used in the GBD 2004 update, which could be classified as ‘moderate and severe’ disability in the disability classes for the GBD study (see Table 5) [29]. The multivariate regression models indicate that DW is positively associated with psychological abnormality, ascites, impaired work capacity, and co-infection by active hepatitis B virus (HBeAg positive), whereas splenectomy is a protective factor for quality of life (see Tables 7 and 8).

Abdominal ultrasonography is considered as a specific and sensitive examination for diagnosis of advanced schistosomiasis [34]. In this study, although 88.8% (191/215) of cases had been subjected to etiologic and symptomatic treatment, more than half (61.1%; 121/198) were detected with hepatic fibrosis of grade III characterized by ‘fish-scale’, ‘turtle-back’, or ‘map-like’ pathognomonic pattern of ultrasonography in parenchyma of the liver, obviously different from post-hepatitis cirrhosis [35]. The enlargement of portal vein is a typical indicator of portal hypertension, which implies the risk of upper gastrointestinal hemorrhage [25], [36]. Referring to normal values obtained from the general population, the mean of inner diameter of the major portal vein was 3.9 mm larger than that of the normal value [25]. Anemia is a common outcome of *Schistosoma* infection and is further aggravated by the emergence of hypersplenism in the advanced stage [5]. It is showed that the mean hemoglobin of advanced cases is far less than that of the normal population (see Table 2).

The EQ-5D is a valid generic questionnaire that is frequently used for describing and measuring health-related quality of life [21], [27], [37], [38]. Our study showed that the impairment rates of advanced cases in each of six health dimensions of EQ-5D+C questionnaire are all significantly higher than those of early-stage chronic cases we had assessed (all *P*<0.001) (see Tables 3 and 4) [16]. Moderate or extreme problems were reported by 55.8% (787/1410) of the chronic cases and by 96.3% (207/215) of the advanced cases. Severe impairments in each of health dimensions contributed considerably to the severe disabilities seen in advanced cases. Activity restriction such as impairment of mobility or self-care is a severe disability, especially for people living in remote areas who are primarily engaged in agriculture. Some characteristics of advanced schistosomiasis – such as long duration, frequent relapse of ascites, impaired work capacity, and worsening family economic status – could induce severe psychological problems [24], [30], [31]. Our study shows that the reported rates of pain or discomfort, or anxiety or depression among advanced schistosomiasis japonica cases are very high, reaching 90.7% and 80.9%, respectively, which explains most of the variation of DW in model 1. Impaired usual activities or work capacity were reported in 87.9% or 92.1% of the patients, which could be explained as the combined effect of anemia, ascites, impaired liver function, splenomegaly, and other complications.

Ascites means a serious impairment and disability affecting a person's ability to work or take part in family and community activities. In the current study, it has been shown that the DW of ascites is 0.135 higher than that of megalosplenia. Compared with megalosplenia type cases, those suffering from ascites manifest themselves with lower quality of life, longer duration of disability, higher reoccurring rates, worse treatment outcomes, and greater overall family burden [24]. In P.R. China, splenectomy, either with or without portosystemic anastomosis, is a general surgical intervention for the treatment of portal hypertension due to hepatosplenic schistosomiasis, which would help to reduce the risk of upper gastrointestinal hemorrhage, cure refractory ascites, recover work capacity, and remove hypersplenism [6].

P.R. China is one of the most highly endemic areas for chronic HBV infection in the world [39]. Superimposed HBV infection has been suggested to play a role in the development of the more severe form of liver disease among cases with schistosomiasis [4], [40]. There is a general agreement that the association of HBV and *S. japonicum* infection are associated with higher morbidity and mortality compared with either infection alone [40], [41]. In this study, 32.8% of advanced cases were found to be positive for HBsAg and HbeAg. Positivity was positively related with DW in advanced schistosomiasis, revealing that there is an active interaction between HBV and schistosome infection.

The current investigation suffers from several limitations that are offered for consideration. First, co-morbidity was inevitably included in this disability assessment. Although hepatitis with clinical features was excluded after a physical examination, four of 198 cases were detected to be HBeAg positive and kept in the statistical analysis. However, given this small number, it is unlikely that exclusion of these four cases would have substantially changed the overall DW of advanced schistosomiasis japonica. Second, a control group (absence of advanced schistosomiasis japonica, matched for age and sex) was not available. The quality of life has been defined as a person's subjective sense of wellbeing, derived from current experience of life as a whole. The use of a VAS, as an approach of psychometrics, allows visualizing, and hence estimating the gap between the ‘real’ health and an ideal, hypothetical stage of health (best imaginable) of the respondents. Hence, the respondent becomes the control of him- or herself [26], [38]. Additionally, it is not always easy to obtain representative population samples of health states associated with a given sequelae, particularly those with a low population prevalence [14]. We had tried to focus our attention on advanced schistosomiasis japonica, and avoided administering a similar questionnaire twice on the chronic cases (early stage). However, in a subsequent study, the current results obtained from patients with advanced schistosomiasis japonica could be readily compared with those obtained from chronic cases reported in our previous work [16] or from other inpatients without schistosomiasis. Third, the general health condition of people is likely to deteriorate as they become older. It should be noted, however that this effect could be offset by a lower health expectation of the elderly [42].

We conclude that uncertainty remains while assessing DWs, particularly for burden estimates due to neglected tropical diseases [13], [43]–[45]. There is a pressing need to incorporate new findings from studies as the one reported here into the current revisions and updates of the GBD study [14], [18], [44]–[47]. Re-evaluation and recalibration of health burden of helminthic parasite infection would highlight the strong potential of integrated parasite control that are likely to go hand-in-hand with efforts for poverty alleviation [13], [28], [48]. We therefore believe that the results presented here provide valuable data for a revision of the local, regional, and global burden of schistosomiasis, as well as for evidence-based decision making in P.R. China's national schistosomiasis control program.
